# Supplementary material for: Metagenomic profiling of ticks: Identification of novel rickettsial genomes and detection of tick-borne canine parvovirus
Source: PLoS Negl Trop Dis. 2019 Jan 14;13(1):e0006805. doi: 10.1371/journal.pntd.0006805 (PMC6347332; doi:10.1371/journal.pntd.0006805)
Supplement: S3 Table — (DOCX) [file pntd.0006805.s003.docx]

|  | Tick species | | | Host species | | |
| --- | --- | --- | --- | --- | --- | --- |
| Tick name | Query ID; Query name | Pairwise identity | Hit length | Query ID; Query name | Pairwise identity | Hit length |
| Jericho camel tick 2.1 | AJ437061; *Hylomma dromedarii* mitochondrion | 100 | 718 |  |  |  |
| Hebron dog tick 1.1 | KF251029; mitochondrial *R. sanguineus* KVI-Rs3 cytC | 99.4 | 626 | KU290572; *Canis lupus familiaris* | 100 | 1560 |
| Nablus sheep tick 1.1 | KY606298; *R. turanicus* isolate 35C | 99.2 | 853 |  |  |  |
| Nablus sheep tick 2.1 | KU214592; *R. sanguineus* | 99.1 | 853 | XM_012105050; *Ovis aries* | 100 | 303 |
| Nablus sheep tick 3.1 | KY606298; *R. turanicus* isolate 35C | 99.2 | 852 |  |  |  |
| Tubas sheep tick 4.1 | KY606298; *R. turanicus* isolate 35C | 99.3 | 855 |  |  |  |
| Tubas sheep tick 4.2 | KY606298; *R. turanicus* isolate 35C | 99.2 | 861 |  |  |  |
| Ramallah dog tick 1.1 | KY364906 ;*H. concinna* mitochondrian | 87.7 | 1505 |  |  |  |
| Ramallah dog tick 1.2 | KY364906; *H. concinna* mitochondrian | 87.8 | 1515 | KU290572; *Canis lupus familiaris* | 100 | 342 |
| Nablus dog tick 1.1 | KF251029 ;mitochondrial *R. sanguineus* KVI-Rs3 cytC | 99.4 | 519 |  |  |  |
| Nablus dog tick 1.2 | KF251029; mitochondrial *R. sanguineus* KVI-Rs3 cytC | 99.2 | 515 | KU290572; *Canis lupus familiaris* | 100 | 496 |
| Tubas dog tick 2.1 | KF251029; mitochondrial *R. sanguineus* KVI-Rs3 cytC | 99.2 | 516 | KU290572; *Canis lupus familiaris* | 100 | 1556 |
| Tubas dog tick 3.1 | MH094482; *Rhipicephalus cf. sanguineus* | 99.3 | 835 |  |  |  |
| Tubas dog tick 3.2 | KY678135; *R. sanguineus* Rsang2 cytC | 99.9 | 770 | KX379529; *Canis lupus familiaris* | 100 | 1558 |
