# Supplementary material for: Metagenomic profiling of ticks: Identification of novel rickettsial genomes and detection of tick-borne canine parvovirus
Source: PLoS Negl Trop Dis. 2019 Jan 14;13(1):e0006805. doi: 10.1371/journal.pntd.0006805 (PMC6347332; doi:10.1371/journal.pntd.0006805)
Supplement: S8 Table — (DOCX) [file pntd.0006805.s008.docx]

| Tick name | Reference used | Number of reads mapped | % of query covered |
| --- | --- | --- | --- |
| Jericho camel tick 2.1 | *F. persica*; CP013022 | 15,031 | 48 |
| Hebron dog tick 1.1 | *Coxiella* endosymbiont; CP001612 | 1385 | 8 |
| Nablus sheep tick 1.1 | *R. africae*; CP001612 | 238,744 | 97 |
| Nablus sheep tick 2.1 | *Coxiella* endosymbiont (CP01126) | 16,624 | 70 |
| Nablus sheep tick 3.1 | *Coxiella* endosymbiont (CP01126) | 340,379 | 100 |
|  | *R. massiliae;* GCA_000016625 | 159,440 | 99 |
| Tubas sheep tick 3.1 | *Coxiella* endosymbiont (CP01126) | 5385 | 35 |
| Tubas sheep tick 3.2 | A*. ovis*; CP015994 | 28,027 | 48 |
| Ramallah dog tick 1.1 | *Coxiella* endosymbiont; CP001612 | 5418 | 0.01 |
| Ramallah dog tick 1.2 | *Coxiella* endosymbiont; CP001612 | 13402 | 0.1 |
| Nablus dog tick 1.1 | *Coxiella* endosymbiont; CP001612 | 10,367 | 30 |
| Nablus dog tick 1.2 | *Coxiella* endosymbiont (CP01126) | 7561 | 48 |
| Tubas dog tick 2.1 | *Coxiella* endosymbiont; CP001612 | 3867 | 9 |
| Tubas dog tick 3.1 | *Coxiella* endosymbiont (CP01126) | 16,624 | 70 |
| Tubas dog tick 3.2 | *Coxiella* endosymbiont (CP01126) | 51,017 | 69 |
