# Supplementary material for: Metagenomic profiling of ticks: Identification of novel rickettsial genomes and detection of tick-borne canine parvovirus
Source: PLoS Negl Trop Dis. 2019 Jan 14;13(1):e0006805. doi: 10.1371/journal.pntd.0006805 (PMC6347332; doi:10.1371/journal.pntd.0006805)
Supplement: S9 Table — (DOCX) [file pntd.0006805.s009.docx]

|  | CRs Tubas | | | | CRt Nablus |
| --- | --- | --- | --- | --- | --- |
| Gene name | NCBI id | Hit length | % pairwise identity | % query coverage | %pairwise identity |
| 16S rRNA | HQ116458 | 1140 | 99.9 | 100 | 96.2 |
| rpoB | KP985338 | 491 | 99.4 | 100 | 99.2 |
| rpoB | KP985339 | 491 | 99.4 | 100 | 99.2 |
| Heat shock protein | KP985425 | 560 | 99.6 | 100 | 98.2 |
| Heat shock protein | KP985426 | 560 | 99.6 | 100 | 98.2 |
| groEL | KP985520 | 578 | 99.5 | 100 | 98.8 |
| groEL | KP985521 | 578 | 99.5 | 100 | 98.8 |
| 23S rRNA | KP994750 | 390 | 99.7 | 100 | 99.2 |
| 23s rRNA | KP994751 | 390 | 99.7 | 100 | 99.2 |
| 16S rRNA | KP994843 | 1209 | 99.5 | 100 | 96 |
| 16S rRNA | KP994844 | 1209 | 99.5 | 100 | 96 |
| 16S rRNA | KU892220 | 1359 | 99.9 | 100 | 96.6 |
| dnaK | KY678156 | 414 | 96 | 100 | 95.6 |
| rpoB | KY678177 | 441 | 99.5 | 100 | 99.2 |
