# Supplementary material for: Metagenomic profiling of ticks: Identification of novel rickettsial genomes and detection of tick-borne canine parvovirus
Source: PLoS Negl Trop Dis. 2019 Jan 14;13(1):e0006805. doi: 10.1371/journal.pntd.0006805 (PMC6347332; doi:10.1371/journal.pntd.0006805)
Supplement: S3 Data — (DOCX) [file pntd.0006805.s012.docx]

| Biosample ID | Sample name | Tick name |
| --- | --- | --- |
| SAMN10176555 | RDT1.1 | Ramallah Dog Tick 1.1 |
| SAMN10176556 | NDT1.2 | Nablus Dog Tick 1.2 |
| SAMN10176557 | TDT1.2 | Tubas Dog Tick 1.2 |
| SAMN10176558 | JCT2.1 | Jericho Camel 2.1 |
| SAMN10176559 | RDT1.2 | Ramallah Dog Tick 1.2 |
| SAMN10176560 | NDT1.1 | Nablus Dog Tick 1.1 |
| SAMN10176561 | TDT2.1 | Tubas Dog Tick 2.1 |
| SAMN10176562 | TDT3.1 | Tubas Dog Tick 3.1 |
| SAMN10176563 | NST1.1 | Nablus Sheep Tick 1.1 |
| SAMN10176564 | NST2.1 | Nablus Sheep Tick 2.1 |
| SAMN10176565 | TST3.1 | Tubas Sheep Tick 3.1 |
| SAMN10176566 | HDT1.1 | Hebron Dog Tick 1.1 |
| SAMN10176567 | NST3.1 | Nablus Sheep Tick 3.1 |
| SAMN10176568 | TST3.2 | Tubas Sheep Tick 3.2 |
