# Supplementary material for: Metagenomic profiling of ticks: Identification of novel rickettsial genomes and detection of tick-borne canine parvovirus
Source: PLoS Negl Trop Dis. 2019 Jan 14;13(1):e0006805. doi: 10.1371/journal.pntd.0006805 (PMC6347332; doi:10.1371/journal.pntd.0006805)
Supplement: S1 Fig — (PDF) [file pntd.0006805.s013.pdf]

a)

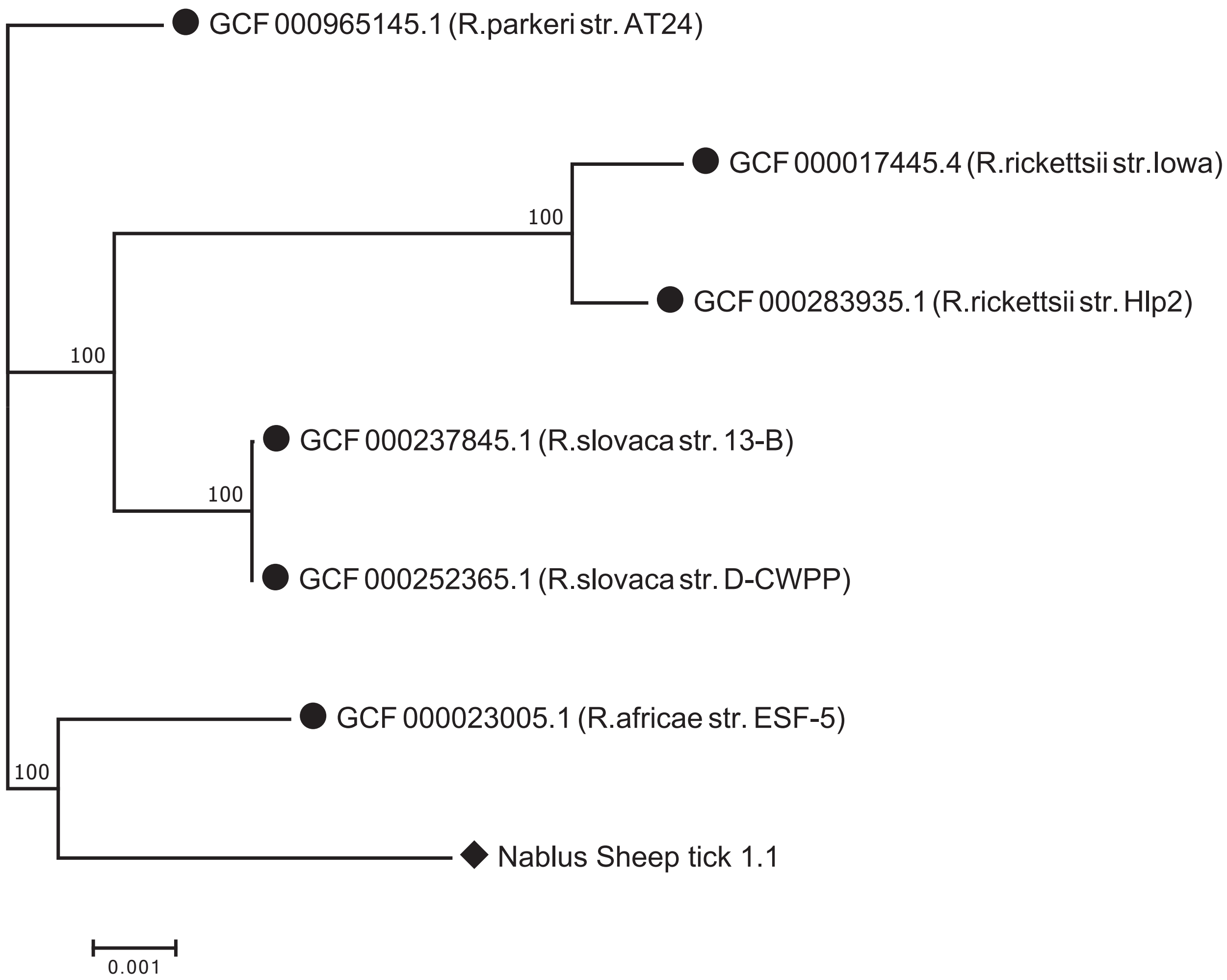

b)

SNP distance matrix

	R.slovaca str. D-CWPP	R.rickettsii str. Hlp2	R.parkeri str. AT24	R.rickettsii str.Iowa	R. slovaca str. 13-B	R.africae str. ESF-5
R.rickettsii str. Hlp2	11683					
R.parkeri str. AT24	6805	13900				
R.rickettsii str. Iowa	12265	3249	14469			
R.slovaca str. 13-B	18	11691	6813	12273		
R.africae str. ESF-5	8592	15541	7335	16097	8584	
Nablus sheep tick 1.1	11450	18338	10214	18904	11458	10593
